# Predicting wrist kinematics from motor unit discharge timings for the control of active prostheses

**DOI:** 10.1186/s12984-019-0516-x

**Published:** 2019-04-05

**Authors:** Tamás Kapelner, Ivan Vujaklija, Ning Jiang, Francesco Negro, Oskar C. Aszmann, Jose Principe, Dario Farina

**Affiliations:** 1 Institute of Neurorehabilitation Systems, University Medical Center Göttingen, Göttingen, Germany; 2 Department of Electrical Engineering and Automation, Aalto University, Espoo, Finland; 3 Department of Systems Design Engineering, University of Waterloo, Waterloo, Canada; 4 Department of Clinical and Experimental Sciences, University of Brescia, Brescia, Italy; 5 Christian Doppler Laboratory for Restoration of Extremity Function and Division of Plastic and Reconstructive Surgery, Department of Surgery, Medical University of Vienna, Wien, Austria; 6 Department of Electrical and Computer Engineering, University of Florida, Gainesville, USA; 7 Department of Bioengineering, Imperial College London, London, UK

**Keywords:** Prosthesis control, EMG decomposition, Neural information, Motor units

## Abstract

**Background:**

Current myoelectric control algorithms for active prostheses map time- and frequency-domain features of the interference EMG signal into prosthesis commands. With this approach, only a fraction of the available information content of the EMG is used and the resulting control fails to satisfy the majority of users. In this study, we predict joint angles of the three degrees of freedom of the wrist from motor unit discharge timings identified by decomposition of high-density surface EMG.

**Methods:**

We recorded wrist kinematics and high-density surface EMG signals from six able-bodied individuals and one patient with limb deficiency while they performed movements of three degrees of freedom of the wrist at three different speeds. We compared the performance of linear regression to predict the observed individual wrist joint angles from, either traditional time domain features of the interference EMG or from motor unit discharge timings (which we termed neural features) obtained by EMG decomposition. In addition, we propose and test a simple model-based dimensionality reduction, based on the physiological notion that the discharge timings of motor units are partly correlated.

**Results:**

The regression approach using neural features outperformed regression on classic global EMG features (average *R*^2^ for neural features 0.77 and 0.64, for able-bodied subjects and patients, respectively; for time-domain features 0.70 and 0.52).

**Conclusions:**

These results indicate that the use of neural information extracted from EMG decomposition can advance man-machine interfacing for prosthesis control.

## Background

Myoelectric control methods translate electromyographic (EMG) signals recorded from the residual limb of amputees into commands for prostheses. Thereby time-frequency domain features are used to extract information from the EMG signals about the user’s intent [1]. Current clinical myoelectric control methods use the EMG amplitude as a feature to control one degree of freedom (DoF) at a time, usually with recordings from an antagonistic muscle pair [2]. Recently commercialized pattern recognition algorithms rely on multiple recording sites and classify time-domain (TD) and/or frequency-domain EMG features into movement classes [3]. Lately, regression methods have been proposed that rely on similar features to create a continuous mapping from the muscle space to kinematics, rather than classification into a discrete number of classes [4–7]. Furthermore, a number of studies used features extracted from additional sensors, such as accelerometers, for performing movement classification [8, 9].

Although essentially different in the way that they provide estimates of the user’s intention, all the aforementioned approaches model the EMG signal as colored noise and so to a large extent neglect the underlying processes of signal generation [10]. Despite the fact that these research efforts have been ongoing for decades, they demonstrated limited clinical impact. When considering the physiological EMG generation, the signal can be modeled as the convolutive mixture of (partly correlated) sources, i.e. series of motor unit discharge timings [11]. Therefore, it is possible to decompose the interference EMG to identify the activities of the motor neurons innervating the muscle, i.e. the neural drive to the muscle [12–14]. The estimated neural drive can then theoretically be used as a control signal for prosthetic applications [15].

We previously demonstrated that, in patients who underwent targeted muscle reinnervation (TMR), the use of motor unit discharge timings outperformed global EMG features for pattern recognition [15, 16]. In this study, we hypothesize that it is possible to estimate wrist joint kinematics by regression applied to motor unit activity, based on the relation between motor neuron behavior and muscle force. The estimated wrist joint angles could allow restoration of the natural control through simultaneous activation of multiple DoFs of a prosthesis and eliminate the need for additional efforts in order to return to the neutral position (position control). Specifically, we describe and validate an approach for predicting joint angles for wrist flexion/extension, pronation/supination and ulnar/radial deviation from Motor Unit Action Potential (MUAP) trains, referred to as neural features, and we compare the predictions with those obtained from linear regression on global EMG features.

## Methods

### Subjects

Five normally-limbed men and one woman, aged 24–38 years, participated in the study. Moreover, a 57 years-old man with a transradial amputation that occurred 37 years before the experiment was also recruited. He has been a daily user of a myoelectric prosthesis since the amputation.

### Signal acquisition

**Fig. 1:**
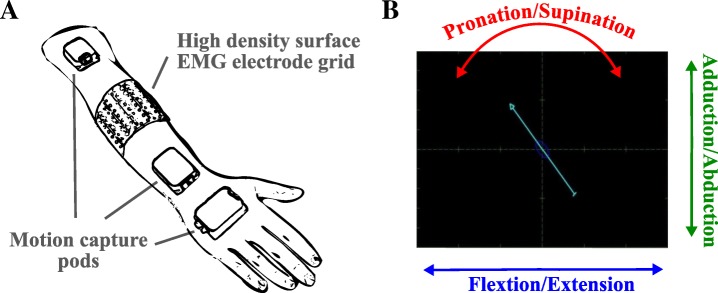
The experimental setup (**a**) and the visual cue provided to the subjects (**b**). Both the high-density EMG electrodes and the motion capture equipment were fixed with elastic bands to prevent displacements. The position and orientation of the pods were used to calculate wrist joint angles. The retrieved wrist trajectories were stored and later used as labels for training and testing of the estimators. Moreover, the current wrist orientation was directly fed back to the participants in order to support them in executing the cued tasks. Changes in wrist joint angles were reflected in the changes in arrow position and orientation, as seen in panel (**b**)

Depending on the anatomy of the subjects, two or three high-density electrode grids (ELSCH064NM3, OT Bioelettronica) were mounted around the dominant forearm (normally limbed subjects) or the residual limb (transradial amputee). The centerline of the grid was at the distal end of the proximal third of the forearm for each subject (Fig. 1A). Each grid consisted of a matrix of 8 × 8 concentric electrodes with a 10 mm diameter and a 10 mm inter-electrode distance. The electrode grids were connected to pre-amplifiers (AD1x64SD5, OT Bioelettronica) and a laboratory EMG amplifier (EMGUSB2, OT Bioelettronica). The EMG signals were recorded in monopolar mode with the sampling frequency of 2048 Hz, 2nd order band-pass filtered between 3 and 900 Hz, and A/D converted to 12 bits. The ground and reference electrodes were placed around the wrists. The ground electrode in the limb-deficient participant was mounted on the lateral elbow epicondyle of the ipsilateral side. A motion capture system (Xsens Technologies B.V., MTx) was used to track wrist kinematics during the performed tasks, and to provide visual feedback to the subjects (Fig. 1). Three pods were attached to the subjects on the dorsal side of the palm, on the wrist and the upper arm just above the elbow. For the subject with limb deficiency, the motion capture equipment was mounted on the contralateral arm. During the experiments, subjects were seated comfortably with their arms relaxed in the neutral position at the side of the body pointing downwards with no additional constrains to any of the joints.

### Experiment procedures

The subjects performed movements of one DoF at a time guided by a visual cue (Fig. 1B). Horizontal movements of an arrow shown on a computer screen corresponded to flexion/extension, vertical movements to adduction/abduction, and rotation to pronation/supination. Visual feedback on the current wrist position was provided by a second arrow. Subjects were instructed to match the two arrows.

For each DoF, the cue prescribed a triangular trajectory at constant speed for both directions of the DoF and the full range of movement. One run consisted of three of these trials for each DoF (random order across DoFs and trials). The subjects performed three runs at three speeds, corresponding to a duration of the ramps of 5 s (slow speed), 2.5 s (medium speed), and 1 s (high speed). The subject with limb deficiency was instructed to perform the movements in a mirrored fashion with both limbs concurrently. Otherwise, the procedures were the same as for the normally-limbed subjects.

### EMG feature extraction

In addition to the analogue filtering, the EMG signal was digitally band-pass filtered using a zero-phase filter of the 5th order with cut-off frequencies 20 Hz and 500 Hz, as commonly used in the myocontrol literature [17]. Signals were visually inspected and noisy channels, which occurred rarely (< 5 channels per recording), were excluded. Then, as recommended [18], the signal was windowed at 100 ms intervals, with 10 ms of overlap resulting in the new feature vector being obtained each 90 ms. The following time-domain features were calculated for each window across all considered channels: root mean square, slope sign changes, zero crossings, and waveform length [3]. The necessary threshold parameters were selected manually for each subject based on visual inspection. The selected values were on average below 10% of the full scale of the amplified signal, and were similar for all subjects. Principal Component Analysis (PCA) was then performed on the extracted feature space containing all trials considered for the controller training, so that the resulting principal components of the features retained 98% of the original variance, as it was previously suggested [19]. This reduced-dimensionality time-domain signal description will be referred to as the TD feature set.

### Neural feature extraction

#### EMG decomposition

The band-pass filtered EMG signals (20 Hz to 500 Hz) were decomposed offline using a convolutive blind source separation algorithm, previously described [12]. The algorithm provides estimates of the time of discharge of a group of motor neurons innervating the muscle (motor neuron spike trains). To maximize the number of decomposed spike trains, EMG signals recorded during activation of individual DoFs were decomposed separately. Thus the algorithm was blinded and unbiased by the fact that some units were active across multiple DoFs. To identify the motor units that were active during tasks of more than one DoF, the waveforms of the motor unit action potentials were compared by cross-correlation. Action potentials identified in different trials were deemed to be generated by the same motor unit if their cross-correlation was > 0.8, as suggested in [20]. The correlation was computed only for channels of the grid with the waveform peak amplitude exceeding the baseline noise standard deviation by 25% for at least one of compared waveforms.

The Decomposed Spike Count (DSC) feature set consisted of the number of firings of each decomposed motor unit in 100 ms intervals, with 10 ms of overlap, as for the TD features. To include the information that was not extracted by the decomposition, features of the residual EMG were also included in the DSC feature set. The residual was computed as the difference between the recorded EMG and the EMG explained by the decomposed spike trains, reconstructed using spike-triggered averaging [21]. The features from the residual EMG were extracted as described in the section “EMG feature extraction”.

#### Model-based dimensionality reduction

EMG decomposition is imperfect, as there are errors in spike identification [22–24]. These errors cannot be corrected by manual editing in an online application. On the other hand, the motor neuron activity is partly correlated [25] and this physiological correlation can be used to mitigate for decomposition errors.

**Fig. 2:**
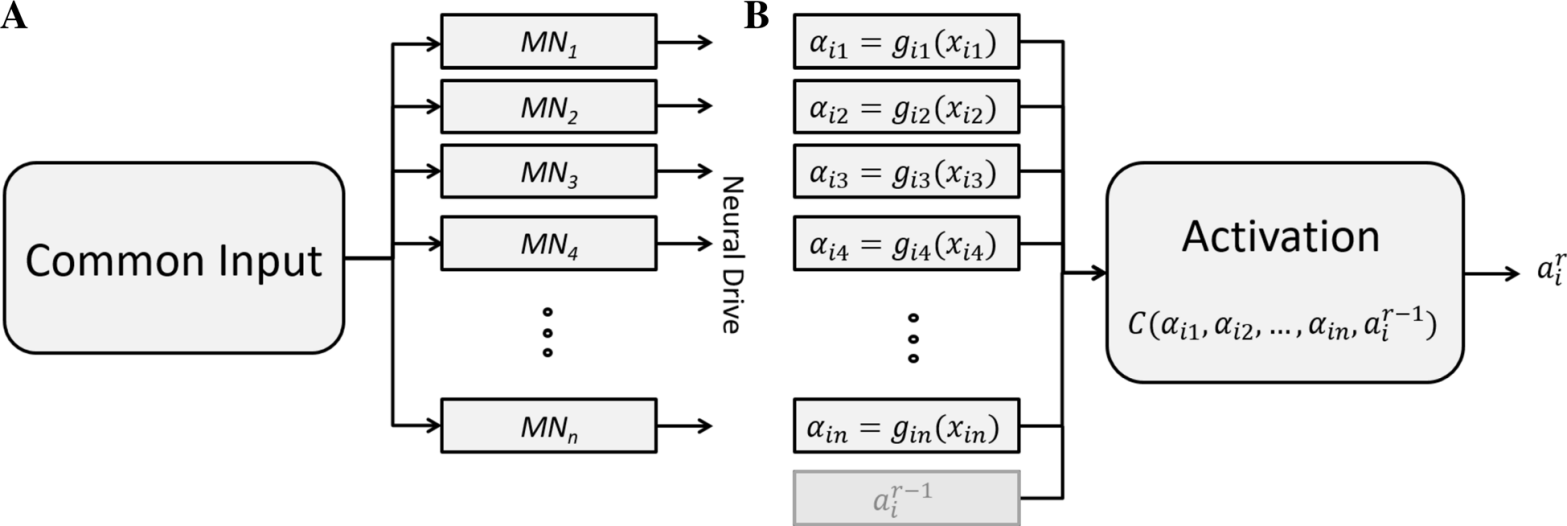
The proposed dimensionality reduction model (**b**) inspired by the physiological model (**a**). Each motor neuron *j* in a pool *i* receives an input *α*_*ij*_ from the central nervous system, which determines the motor unit spike train *x*_*ij*_. The input to the motor neuron is partly common to the other motor neurons in the pool and is associated to the spike train by the function *g*_*ij*_(∙). After estimation of the functions *g*_*ij*_(∙), the synaptic inputs of the motor neurons are used to extract one activation signal ${a}_i^r$ in the time processing window *r* that reflects the common input. The final activation is also obtained by combining in its estimation the activation at the previous processing interval *r* − 1 (${a}_i^{r-1}$) to promote smoothness

Motor neurons in the same pool or across synergistic muscles share a relatively large proportion of their synaptic input [26–28]. Therefore, the discharge timings of each motor unit not only carry information on muscle force, but are also correlated to the activity of other motor units (Fig. 2A).

The relation between the input received by each motor neuron *j* in a pool *i* and the resulting spike train *x*_*ij*_ of the motor unit was modelled by a function *g*_*ij*_(∙):

$$ {\alpha}_{ij}={g}_{ij}\left({x}_{ij}\right) $$

We assume that the input *α*_*ij*_ is associated to the wrist kinematics and can therefore be identified from the wrist joint angles. For this purpose, *g*_*ij*_(∙) was approximated as a linear function [29] and estimated from the spike train using robust linear regression (weighted least squares regression with the bi-square weight function) between the spike train and the joint angle from the training set. For this estimate, each motor unit was associated to the DoF with the highest correlation between the DoF activation and the motor unit spike train. The estimated *α*_*ij*_ for different motor units are similar but not identical because part of the input is not common and because of the presence of decomposition errors. For this reason, it is not possible to directly pool together all spike trains. For each processing interval *r*, we therefore combined the individual estimates *α*_*ij*_ to extract a single activation ${a}_i^r$ for the pool of motor units (Fig. 2B). Moreover, to promote smoothness over time, we included the estimate ${a}_i^{r-1}$ at the previous processing interval:

$$ {a}_i^r=C\left({\alpha}_{i1},{\alpha}_{i2},\dots, {\alpha}_{in},{a}_i^{r-1}\right) $$

Among the possible choices of the operator *C*(·), we chose the median value, which introduces a non-linearity in the estimate:

$$ {a}_i^r=\underset{j}{\mathrm{median}}\left({\alpha}_{i1},{\alpha}_{i2},\dots, {\alpha}_{in},{a}_i^{r-1}\right) $$

**Fig. 3:**
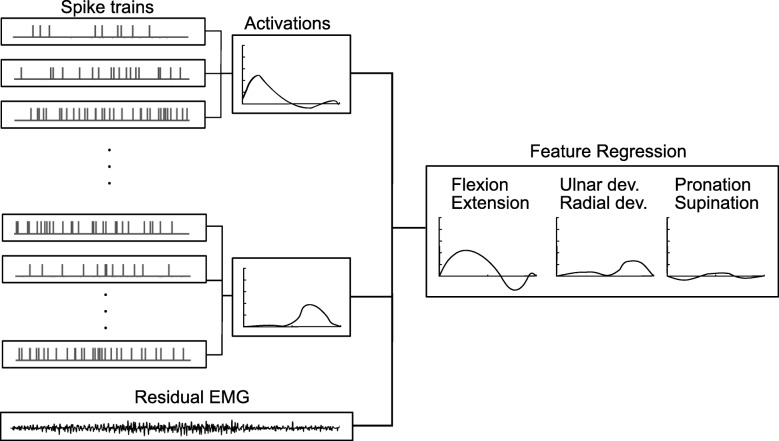
Regression of neural features. The EMG was decomposed into motor unit action potentials, which were grouped according to their correlation (see text), and used to estimate activations, as shown in Fig. 2. The activations and the residual EMG features were then used to predict the wrist DoF angles

Finally, the estimated activations ${a}_i^r$ for each DoF together with the TD features of the residual EMG were used as neural features at the input of the final linear regression (Fig. 3). Therefore, the model presented in Fig. 2 is a signal processing step before regression, which converts discharges of motor neuron populations into activations, reducing the dimensionality of the data.

#### Linear regression of TD and neural features

A simple linear regression [30] was applied for the final estimates of commands (Fig. 3). During the training of the regressor, the training data contained the information on all individual DoFs. Once the training stage was completed, the weights were fixed throughout the testing phase. The regressed estimates from the testing data were then continuously derived across all three DoFs. The regression was applied to both the TD and neural features for comparison. In both cases, the median value of three consecutive outputs of the linear regression was used as the final estimate, similarly to a majority vote approach for classification.

Three-fold cross-validation was performed to assess the robustness of the system. To quantify the performance, *R*^2^ [31] has been used as a measure of goodness of estimated joint angles from the selected features with respect to the actual recorded angles. This metric has been specifically chosen since it accounts for the different ranges of motion of individual DoFs [32]. In each fold, for each subject, the testing data was randomly selected as one ramp of each DoF and was used to evaluate the system trained on the remaining two ramps. This was repeated three times until all data were tested at least once. Three-fold cross-validation was then done 10 times with different combinations.

#### Comparison with other neural feature sets

**Fig. 4:**
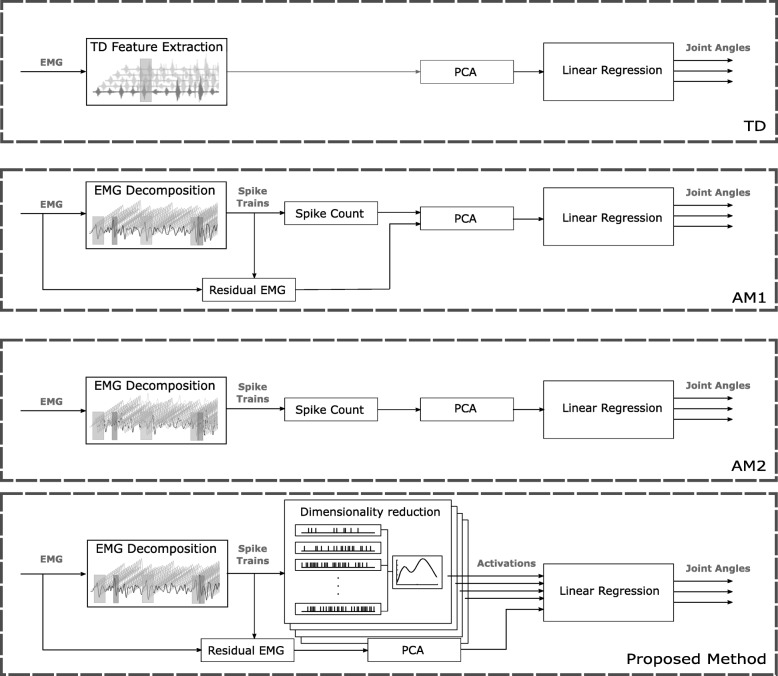
Block diagram of the processing steps for each of the compared features. The top panel shows processing steps for the regression based on Time Domain (TD) features. The middle two panels describe AM1 and AM2 feature regression. The main difference between the two is the inclusion of the residual EMG in addition to Decomposed Spike Count (DSC). The bottom most panel shows the proposed method which includes the model-based dimensionality reduction. It should be noted that in all cases the PCA was applied to the feature space to retain 98% of the variance

In addition to the TD features, the proposed neural approach was also compared with two other feature sets (Fig. 4). The first (indicated as AM1 in the following) comprised the DSC and EMG residual without the model-based dimensionality reduction described previously. The second (AM2) comprised only the DSC without model-based dimensionality reduction and without the residual EMG features. For AM1 and AM2, PCA was applied to the feature space to retain 98% of the variance, as for the TD features.

#### Statistical analysis

Mean ± standard deviation of *R*^2^ was used as descriptive statistics and ANOVA was applied to assess differences in performance between features. First, a full ANOVA model was employed with all interactions between the fixed-level factors “Feature” and “Ramp Duration”, and the random factor “Subject” with levels A1-A6 by which we have anticipated possible natural variation in human data. Differences in features only were analyzed using one-way ANOVA with repeated measures with the constant factor “Feature”, for each subject and ramp duration separately. The performed post-hoc tests were conducted using Bonferroni’s correction considering six pairwise comparisons between the four feature sets (TD, AE1, AE2, and the proposed method). Significance was reported at *p* < 0.05. The subject with limb deficiency (D1) was not included in the statistical analysis and only descriptive results are reported for this subject.

## Results

### EMG decomposition

**Fig. 5:**
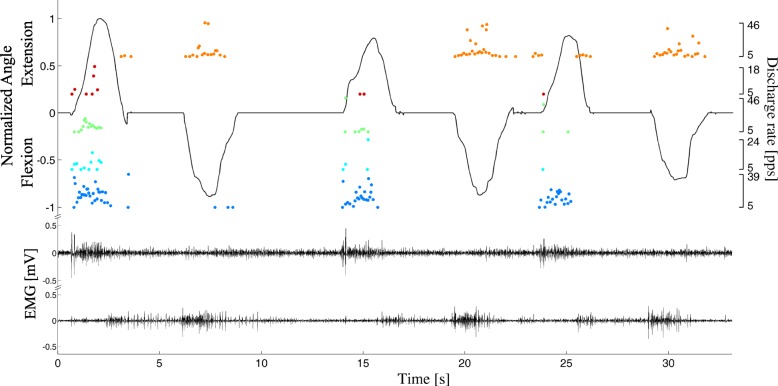
Representative example of EMG decomposition during voluntary contractions. Only two EMG channels are shown for clarity (lower traces). The recorded wrist flexion/extension angle is shown in black (upper trace), and a representative subset of decomposed spike trains is represented as dots, whose values indicate instantaneous discharge rates (right axes). The full automatic decomposition introduced errors in spike identification, including missed spiking activity (e.g., third extension). In this example, only one DoF is depicted for clarity and the steady kinematic output during rests between motions is a result of sensors’ intrinsic inertial properties [43]

All high-density EMG signals recorded during the contractions could be decomposed using the blind source separation algorithm (an example is reported in Fig. 5). Table 1 shows the number of active motor units during each DoF, including motor units that were active in multiple DoFs. The number of decomposed motor units decreased with the ramp duration.

**Table 1:** Number of decomposed motor units per each DoF

Ramp duration		DoF1	DoF2	DoF3	DoF1 + DoF2	DoF1 + DoF3	DoF2 + DoF3	Total
Full movement range	1 s	38.4 ± 10.4 (25)	33.1 ± 9.2 (16)	26.0 ± 7.5 (16)	1.4 ± 1.2 (0)	0.7 ± 0.9 (2)	2.0 ± 1.7 (1)	101.8 ± 25.3 (57)
	2.5 s	24.3 ± 10.7 (11)	22.3 ± 6.8 (18)	23.7 ± 6.9 (25)	2.4 ± 1.4 (0)	1.3 ± 1.3 (2)	2.3 ± 1.3 (3)	76.3 ± 24.1 (54)
	5 s	15.0 ± 2.8 (11)	16.3 ± 6.3 (11)	16.0 ± 4.6 (20)	3.0 ± 3.2 (4)	1.3 ± 0.9 (2)	3.0 ± 1.4 (4)	54.6 ± 11.1 (42)

Mean and standard deviation of the number of decomposed motor units, according to the DoF activations during which they were active. The last column indicates the mean number of all decomposed units per subject. Numbers in brackets indicate values for the subject with a limb deficiency

### Statistical evaluation

**Fig. 6:**
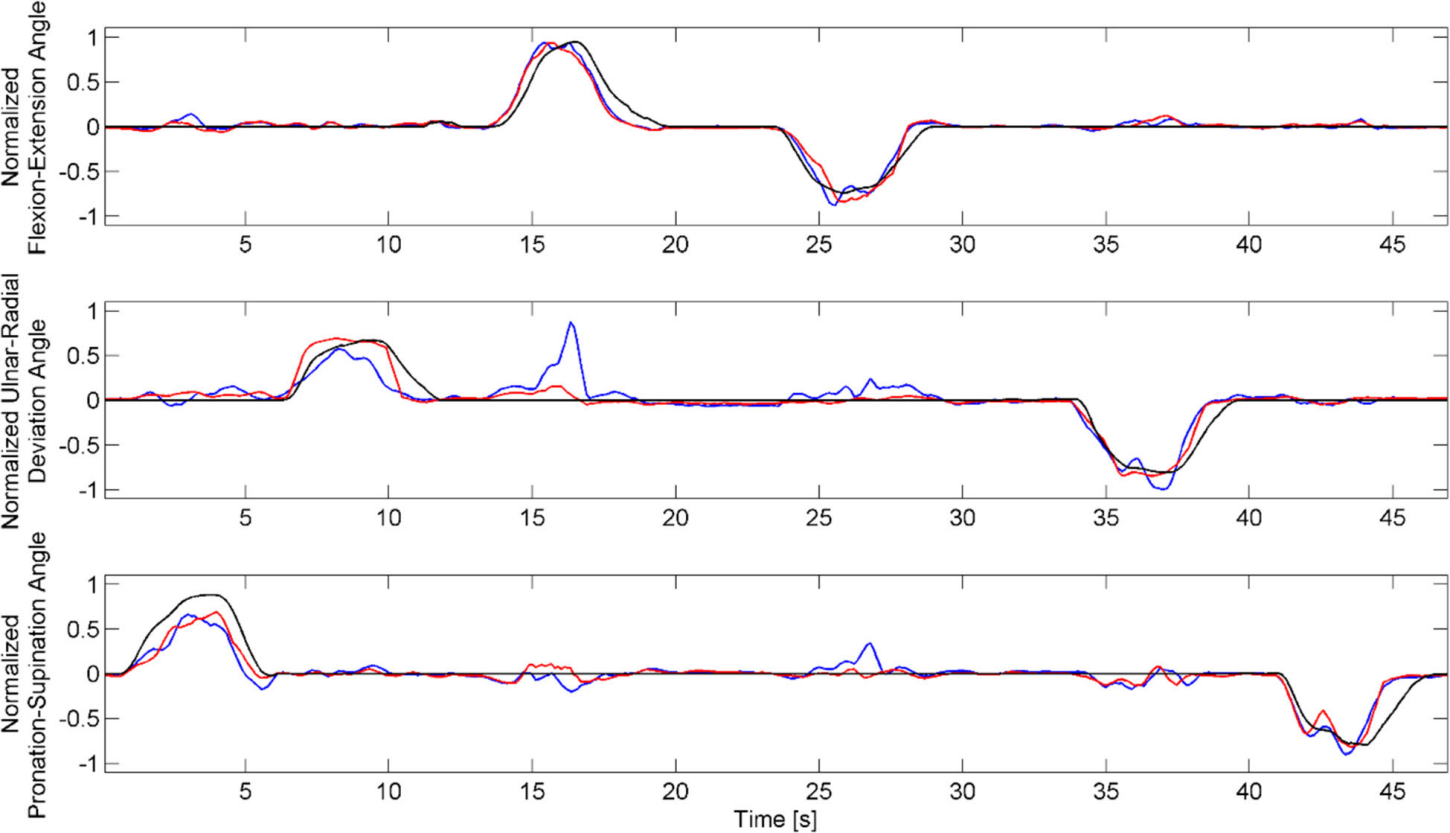
An example of the regression results. TD (blue lines) and neural (red lines) feature sets are compared with the measured kinematics of the subject (black lines). The order of the attempts was randomized during the experiment

The full ANOVA detected statistically significant effects between Features (*p* = 0.0025) and a significant three-way interaction (*p* < 0.001). Fixing the ramp duration and performing two-way ANOVAs, we found that while the significant effect of features remained present in all comparisons (*p* < 0.01), there were significant two-way interactions between the factors “Subject” and “Feature”, across all levels. Therefore, the feature performance for each subject and ramp duration was analyzed separately, as described in the Methods. The post-hoc tests identified statistically significant differences between the TD and the neural feature sets in all subjects and ramp durations, with an average *R*^2^ of 0.77 for the neural features and 0.70 for TD. Similarly, the average *R*^2^ value of subject D1 improved from 0.52 to 0.64 with the proposed method. Differences between Ramp-Durations were not statistically significant. An example of the regression results is shown in Fig. 6.

### Comparison with other neural feature sets

**Fig. 7:**
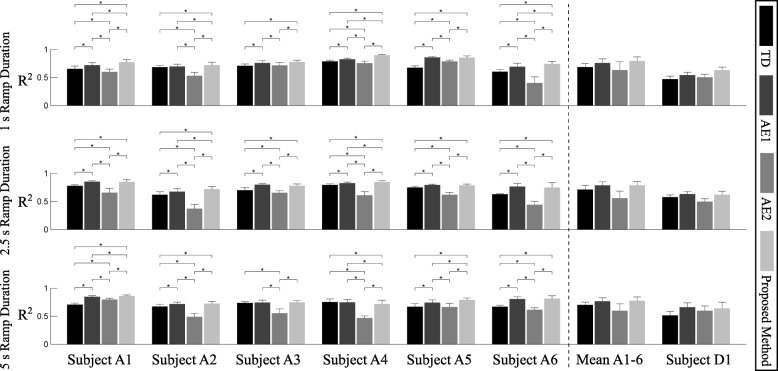
Performance comparison of feature sets at the full range of movement for all subjects and ramp durations. Bars with asterisks indicate statistically significant differences (*p* < 0.05). Note that subjects were treated as a random factor with multiple levels and that Subject D1 was not included in the statistical comparisons. In addition, while the post-hoc analysis indicated statistically significant differences between the TD and the neural feature sets in all subjects and ramp durations, no statistical difference was found between the ramps

We repeated the statistical analysis including the two additional neural feature sets AM1 and AM2. The ANOVA detected statistically significant effects of Features (*p* = 0.0025), significant two-way interaction between the factors Subject-Ramp Duration (*p* < 0.001) and Subject-Feature (*p* = 0.02), as well as a significant three-way interaction (p < 0.001). The post-hoc analysis showed that the proposed methods significantly outperformed both AM1 and AM2 in most cases, and never underperformed them significantly (Fig. 7). As for the proposed method, AM1 also provided an improvement over TD consistently for all conditions.

## Discussion

We compared automatically decomposed motor unit spike trains to traditional EMG features in terms of linear regression performance in myoelectric wrist control. Our primary finding is that spike trains outperformed interference EMG features.

### EMG decomposition

The number of decomposed spike trains depended on the ramp duration (Table 1), so that more spike trains were identified at higher speeds than at lower ones. This indicates that at least in some phases of the movement at higher speeds the contraction level was higher, and thus more motor units were recruited within the pick-up area of the surface electrodes. We also observed that the muscle activations during the tasks were selective, i.e. there were just a few motor units detected in more than one DoF (Table 1). These units have probably been activated as a part of neural control strategies and had an influence on the wrist joint stiffness. The limitation of the applied regressor is that it interpreted these as concurrent activation of DoFs (Fig. 6).

### Feature performance

Neural information extracted from MUAP trains showed better regression performance than traditional TD features, for both able-bodied subjects and a subject with limb deficiency. The tests using the alternative methods showed that both the inclusion of the residual EMG and the proposed dimensionality reduction contributed to the achievement of superior performance.

One plausible reason for the relatively low performance of purely spike-based features is the imperfect decomposition, since including the residual information outperformed traditional features consistently. Additionally, the matching of the MUs across trials was done using a fixed threshold value, which could potentially benefit from case-specific statistical optimization. Another possibility is the non-linear relation between motor unit spike trains and wrist kinematics [33]. Nevertheless, the observation that the proposed method as well as AM1 outperformed TD indicates that spike trains do carry additional information that could not be extracted with traditional features, even for such high channel numbers.

The overall best performing feature set was the proposed model-based neural set (Fig. 7), although a significant advantage resulting from the model-based dimensionality reduction was only observed at the shortest ramp duration (the fastest speed). This is likely due to the decomposition being less accurate for faster contractions, in which case a model-based approach could recover more information. At the same time, TD might benefit from more careful DoF-wise channel selection in addition to PCA. At this stage, the computational load required for extracting neural features is much greater compared to the TD features. The implementation and results presented in this study aimed at a rigorous testing of the concept, to prove the feasibility of the neural approach. Future work should explore online controllers and test their clinical validity with the focus on the implications of the observed increase in offline performance.

The linear regression on spike trains (AM2) was not sufficient to achieve *R*^2^ performance superior to TD. Regression on spike trains including the residual EMG (AM1), however, outperformed traditional features, although the proposed model-based approach further improved performance. In particular, the model-based approach made the most out of the larger population of decomposed MUs available during the shortest ramp duration. This indicates that the proposed physiologically inspired dimensionality reduction method partly counteracted decomposition inaccuracies.

### Dependence on movement speed

There were no statistically significant differences in regression performance between ramp durations. This was an unexpected finding since the number of spike trains, and therefore decomposition complexity increases with movement speed. The significant three-way interaction showed that the effect of ramp duration on performance was subject-specific, indicating that multiple factors influenced regression in addition to decomposition complexity. One confounding factor is the biased sample of the motor units detected by decomposition. Because higher threshold units tend to have action potentials with greater energy than lower threshold units, the sample of decoded units mainly comprises of high threshold units, for which the decomposition and waveform comparison task are more accurate [34]. It is also possible that the action potential shape of some lower threshold units was considerably changed due to muscle motion relative to the electrode, resulting in these units not being detected by the decomposition. Moreover, recruitment threshold may vary with contraction speed [35] which may also negatively influence regression performance. Other confounding factors include the variability in subject anatomy and the properties of the tissue layers between the muscle fibres and the electrodes. These factors influence decomposition accuracy in a subject-specific manner, which might have translated to differences in regression performance.

### Limitations

The main limitation of the study is that we used an offline automatic EMG decomposition method, which is not invariant to the movements of muscles relative to the skin surface since it has been developed for low to medium force isometric contractions and has been shown to be only partly effective for dynamic contractions [36]. We also do recognize that the improvements in the offline control do not necessarily result in the increase of clinical scores [37]. Based on the statistically significant improvement in the offline scores, it is indeed difficult to conclude how beneficial the observed increase in clinical performance will be. However, in this study, we aimed to investigate whether the information gained from EMG decomposition can in principle benefit myoelectric control. Moreover, the presented evaluation is not dependent on the data acquisition method, and can be used with any method for extracting spike trains of motor unit populations, including future online EMG decomposition algorithms of surface or intramuscular EMG [38], as well as spike sorting from other signals such as peripheral nerve recordings [39–41]. It should also be noted that an online implementation of the method used here is feasible [42], and it is also possible to implement an MU tracking algorithm [20] that can provide continuous information on the activity of the relevant MUs while at the same time reducing the computational time needed for signal decomposition.

Another limitation is that we only included single DoF contractions. Although we acknowledge the importance of simultaneous control of multiple DoFs, the present study on single DoFs is a necessary first step for future developments of multi-DoF control based on motor unit activity. Similarly, we have only conducted tests on the tasks that the estimators were familiar with, while additional investigation on handling spurious activity originating from untrained DoFs will be done in future work. Finally, the inclusion of a subject with limb deficiency showed the feasibility of motor unit recordings and regression in the target population of prosthesis users, but these data are not sufficient to demonstrate general clinical applicability or to make more general claims about the observed performance. Overall, having shown that we are able to precisely regress the recovered neural information we have established a framework for the development of more efficient and ultimately real-world viable control systems.

## Conclusions

Decomposed motor unit spike trains outperformed traditional EMG features when used with linear regression in myoelectric control of the wrist, for both able-bodied subjects and an individual with limb deficiency. A novel dimensionality reduction method based on physiological principles of motor unit behavior showed better overall performance than the other investigated features. We also found that the relationship between regression performance using neural information and movement speed is subject-specific. Based on these results we suggest that the use of neural information extracted from EMG decomposition can advance man-machine interfacing for prosthesis control. We also foresee the extension of the proposed neural model to a neuro-musculoskeletal model in which the linear regressor would be extended to a controller that would account for other parameters of the system, such as inertia. Potentially, the more accurate model of motor neuron outputs would in that case be beneficial.

## Abbreviations

A/D: Analog/Digital
ANOVA: Analysis of Variance
DoF: Degree of Freedom
DSC: Decomposed Spike Count
EMG: Electromyogram
MUAP: Motor Unit Action Potential
PCA: Principle Component Analysis
TD: Time Domain
